# Supplementary material for: The Role of Polygenic Susceptibility on Air Pollution-Associated Asthma between German and Japanese Elderly Women
Source: Int J Environ Res Public Health. 2022 Aug 10;19(16):9869. doi: 10.3390/ijerph19169869 (PMC9407879; doi:10.3390/ijerph19169869)
Supplement: Supplementary file 1 [file ijerph-19-09869-s001.zip › ijerph-1814938-supplementary.pdf]

Supplementary material

‘The role of polygenic susceptibility on air pollution-associated asthma between German and Japanese elderly women’

Sara Kress, Akinori Hara, Claudia Wigmann, Takehiro Sato, Keita Suzuki, Kim-Oanh Pham, Qi Zhao, Ashtyn Areal, Atsushi Tajima, Holger Schwender, Hiroyuki Nakamura & Tamara Schikowski

Content:

Text details 2
1. Air pollution assessment 2
2. Genotyping and summary of genetic data 2
3. Quality control steps and imputation 3
4. Assessments of polygenic risk scores 3
5. Genetic risk score-interaction-training approach 3
6. Definitions of potential confounders 4

Tables 4

Table S1 Information on 128 asthma-related single nucleotide polymorphisms from genome-wide association studies that were summarized by El-Husseini et al. [9]. A total of 107 single nucleotide polymorphisms were included in our calculation of the polygenic risk score. 4

Table S2 Information on 11 asthma-related single nucleotide polymorphisms that were included in our calculation of the polygenic risk score from the genome-wide association study by Ishigaki et al. [10]. 8

Table S3 Description of the study samples, asthma and air pollution exposures in the main and sensitivity analyses 9

Table S4 Gene-environment interaction effects on asthma among German and Japanese women using different adjusted logistic regression models: the main results and results of sensitivity analyses. 10

Figures 12

Figure S1 Effects of air pollution exposure on asthma for each sample-specific interquartile range increase of the polygenic risk score in elderly (a) German never-smoker women and (b) Japanese never-smoker women. 12

References 13

Text details

1. Air pollution assessment

SALIA

For SALIA study participants, air pollutant exposures were assigned within the European Study of Cohorts for Air Pollution Effects (ESCAPE) [1,2]. Thereby, 14-day measurements of $PM_{2.5}$ in each cold, warm and intermediate temperature season between October 2008 and November 2009 from 20 monitoring sites were conducted. NO_2 was monitored at 40 sites. With a central reference monitoring site, which measured concentrations of air pollution using the same instruments continuously for a complete year, the values were adjusted for the true long-term average of the observation period. Finally, land-use regression models predicted the air pollution concentrations at the home addresses of each participant at the first follow-up examination. The data quality was examined and assured, e.g. only sites with high-quality data for at least 75% of the days in a year were used.

Shika study

For Shika study participants, daily ambient concentrations of $PM_{2.5}$ and NO_2 between April 2011 and March 2020 were obtained using the Atmospheric Environmental Regional Observation System provided by the Japanese Ministry of the Environment. Local air pollutants concentrations were monitored by the Nanao monitoring station (136.6 °E, 37.2 °N; distance to the central of Shika: 18.8 km). After calculating the annual mean concentration of each pollutant, individual-level exposures to each pollutant were defined as the average of the annual mean concentrations of each air pollutant during the observation periods of each participant.

2. Genotyping and summary of genetic data

SALIA

Genome-wide genotyping was performed in December 2016/ January 2017 in 468 blood and saliva samples and additional in November 2020 in 284 blood and saliva samples using the Axiom Precision Medicine Research Array (Affymetrix, Santa Clara, CA, USA) (GRCh37/hg19) resulting in 871,262 variants. 586 individuals and 410,652 SNPs passed the pre-imputation quality control.

386,710 SNPs passed the quality control of the Michigan Imputation Server. After post-imputation quality control 586 individuals and 7,643,653 SNPs remained.

Shika study

Genome-wide genotyping was performed from 2018 to 2020 in 1,325 blood samples using the Japonica Array v2 [3] (TOSHIBA, Tokyo, Japan) resulting in 659,709 variants. 1,055 individuals and 608,266 SNPs passed the pre-imputation quality control. 598,841 SNPs passed the quality control of the Michigan Imputation Server. After post-imputation quality control 1,055 individuals and 6,567,060 SNPs remained.

3. Quality control steps and imputation

For both cohorts, in the pre-imputation quality control [4] variants on chromosome 0, insert/deletion variants, variants with low minor allele frequency (<0.01), and low call rates (<0.95) were excluded. After that, duplicated individuals, individuals with sex-mismatch, with low call rates (<0.95), with minimal heterozygosity (inbreeding coefficient 0.1), highly related individuals (identity-by-descent analysis with Id.tresh=0.2 and kin.tresh=0.1), as well as individuals belonging to another ethnic group (Tukey's rule based on the 1-10 eigenvectors from principal component analysis), and violations of Hardy-Weinberg ($p<10^{-6}$) were removed. Finally, SNPs that deviate from Hardy-Weinberg equilibrium ($p<10^{-6}$) were removed. Strand designation/ strand flips correction was done.

To find haplotype segments that are shared by study individuals and the HRC r1.1 2016 (GRCh37/hg19), we did a genotype imputation with minimac4 1.5.7 using the Michigan Imputation Server [5].

In post-imputation processing multi-allelic markers and variants with low minor allele frequency (<0.01), as well as variants with low imputation quality ($R^2<0.3$) were excluded [5].

4. Assessments of polygenic risk scores

Study sample-specific polygenic risk scores (PRS), defined as the individual sum of all risk variant alleles, multiplied by the weight of each allele on the risk of asthma from the appropriate GWAS, were calculated using the EBPRS R-package [6]. This novel method is based on the Empirical Bayes Theory and leverages internal effect size distributions to improve prediction accuracy compared to other approaches to PRS calculations. In the PRS calculation, effect sizes for each SNP were estimated by bootstrapping approach with a robust estimation based on both, summary statistics (odds ratio and p-value) from the GWAS as well as the number of study individuals presenting the outcome. Differences in the definition of risk allele between the cohort and the GWAS are considered. Using the estimated effect sizes, the PRS for each study participant was generated (higher values represented a higher risk of asthma).

5. Genetic risk score-interaction-training approach

In the genetic risk score (GRS)-interaction-training approach [7], the sample data was split into a training dataset and a test dataset. The training dataset was used to calculate the weight of each SNP and the test dataset was further used to calculate the GRS and perform the GxE analysis. The weights were gained from the interaction terms between each SNP and the air pollutant exposure using elastic net regression models. Since there may be SNPs that are only important in areas with high air pollution exposure and these SNPs may not be included in association analyses on the outcome alone, the interaction terms were used. The optimal balance of sample sizes between training and test datasets in our study was determined to be 1:2. The sample-specific GRSs were based on all SNPs that are associated with asthma in the specific GWAS and available in the specific cohorts (the same SNPs as in the main models).

6. Definitions of potential confounders

SALIA

The appropriate available potential confounders were selected for the elderly women [8] such as age, height, weight, highest education of the participant or her spouse (low<10 years, high≥ 10 years of education) and smoking history (ever vs. never).

Shika study

Among the data that have been collected in the Shika study, education status and smoking history as well as age, height, and weight, were used. As in the SALIA study, the education status was expressed as a binary value of ≥10 years or <10 years, and the smoking history was expressed as a binary value of ever or never smoked.

Tables

Table S1. Information on 128 asthma-related single nucleotide polymorphisms from genome-wide association studies that were summarized by El-Husseini et al. [9]. A total of 107 single nucleotide polymorphisms were included in our calculation of the polygenic risk score.

					SALIA			
**CHROM**	**rsID**	**POS**	**REF**	**ALT**	**MAF**	**TYPED**	**R2**	**ER2**
1	rs11121240	8894357	A	T	0.42	IMPUTED	0.95	-
1	rs67551275	9356676	C	T	-	-	-	-
1	rs662064	10557251	T	C	0.30	IMPUTED	0.96	-
1	rs6600246	25299426	C	T	0.14	IMPUTED	0.99	-
1	rs541559418	151760522	G	A	-	-	-	-
1	rs11204896	151796742	C	G	0.11	IMPUTED	0.98	-
1	rs12123821	152179152	C	T	0.04	IMPUTED	0.74	-
1	rs61816761	152285861	G	A	0.01	IMPUTED	0.76	-
1	rs4129267	154426264	C	T	0.36	GENOTYPED	1.00	1.00
1	rs1101999	158932555	C	T	-	-	-	-
1	rs4233366	161159147	C	T	0.23	IMPUTED	0.97	-
1	rs1723018	167433420	A	G	0.40	IMPUTED	0.97	-
1	rs1102705	172700868	A	G	-	-	-	-
1	rs6691738	173152036	T	G	0.32	IMPUTED	0.99	-
1	rs2786098	197325908	T	G	0.21	GENOTYPED	1.00	1.00
1	rs6683383	203100504	T	A	0.35	IMPUTED	0.97	-
2	rs13412757	8458080	G	A	0.35	IMPUTED	0.96	-
2	rs3771180	102953617	G	T	0.14	GENOTYPED	1.00	0.99
2	rs34290285	242698640	G	A	0.23	IMPUTED	0.97	-

3	rs115913567	23653570	G	C	-	-	-	-
3	rs35570272	33047662	G	T	0.39	IMPUTED	0.97	-
3	rs1806656	121716171	C	G	0.29	IMPUTED	0.96	-
3	rs7625643	141150026	A	G	-	-	-	-
3	rs7626218	176852038	A	T	0.39	IMPUTED	0.98	-
3	rs519973	187633268	G	A	0.34	GENOTYPED	1.00	0.98
3	rs2030030	187793833	T	C	0.17	IMPUTED	0.97	-
3	rs60946162	188133336	C	T	0.43	GENOTYPED	1.00	0.96
3	rs73196739	188402471	C	T	0.15	IMPUTED	0.98	-
3	rs11715524	195762492	G	A	0.48	IMPUTED	0.95	-
3	rs1684466	196359310	G	A	0.34	IMPUTED	0.88	-
4	rs4833095	38799710	T	C	0.21	GENOTYPED	1.00	1.00
4	rs45613035	123141070	T	C	0.12	IMPUTED	0.97	-
4	rs4380538	123448562	C	T	0.36	IMPUTED	0.98	-
4	rs7686660	144003159	T	G	0.24	GENOTYPED	1.00	1.00
5	rs16903574	14610309	C	G	0.08	IMPUTED	0.84	-
5	rs11742240	35881376	G	T	0.30	IMPUTED	0.99	-
5	rs1588265	59369794	A	G	0.29	GENOTYPED	1.00	1.00
5	rs6893213	110198114	C	T	0.08	IMPUTED	0.97	-
5	rs1837253	110401872	T	C	0.29	GENOTYPED	0.99	0.84
5	rs1438673	110467499	C	T	0.46	GENOTYPED	1.00	0.95
5	rs6894249	131797547	A	G	0.38	IMPUTED	0.99	-
5	rs6871536	131969874	T	C	0.27	GENOTYPED	1.00	1.00
5	rs113010607	132105698	T	C	-	-	-	-
5	rs740474	140925362	C	T	0.39	IMPUTED	0.95	-
5	rs200634877	141529762	NA	AAAAT	-	-	-	-
5	rs449454	141533062	A	G	0.38	IMPUTED	0.97	-
6	rs1233578	28712247	A	G	0.14	GENOTYPED	1.00	1.00
6	rs3190923	31324499	C	G	-	-	-	-
6	rs2507978	31351664	G	A	0.45	IMPUTED	1.00	-
6	rs2855812	31472720	G	T	0.26	GENOTYPED	1.00	1.00
6	rs28895016	31574525	C	T	0.05	IMPUTED	0.99	-
6	rs404860	32184345	T	C	0.14	GENOTYPED	1.00	0.94
6	rs3117098	32358513	G	A	0.32	GENOTYPED	1.00	1.00
6	rs115468973	32544121	C	T	-	-	-	-
6	rs1064713	32546666	G	A	-	-	-	-
6	rs17843604	32620283	C	T	0.48	GENOTYPED	0.99	0.90
6	rs987870	33042880	A	G	0.12	GENOTYPED	1.00	0.99
6	rs3097670	33046752	G	C	0.12	IMPUTED	0.98	-
6	rs10947428	33647058	T	C	0.22	GENOTYPED	1.00	1.00
6	rs1776883	34156444	C	T	0.49	IMPUTED	0.91	-

6	rs28522747	35134729	G	A	0.12	IMPUTED	1.00	-
6	rs9357733	52292136	A	G	0.17	IMPUTED	0.97	-
6	rs58521088	90985198	A	T	0.31	IMPUTED	0.96	-
6	rs55743914	128293562	C	T	0.23	GENOTYPED	1.00	0.90
6	rs6927172	138002175	C	G	0.21	IMPUTED	0.99	-
7	rs149317277	20423923	NA	G	-	-	-	-
7	rs6461503	20560996	T	C	0.45	IMPUTED	0.94	-
7	rs4722758	28156606	C	G	0.22	IMPUTED	0.93	-
7	rs6967330	105658451	G	A	0.15	GENOTYPED	0.98	0.77
8	rs7009110	81291879	T	C	0.37	GENOTYPED	1.00	1.00
8	rs3019885	118025645	T	G	0.41	IMPUTED	0.95	-
8	rs13277355	128777719	A	G	0.26	IMPUTED	0.92	-
9	rs343478	6051399	G	A	0.44	IMPUTED	0.98	-
9	rs1342326	6190076	A	C	0.16	GENOTYPED	1.00	0.99
9	rs72721168	27308288	A	C	0.03	IMPUTED	0.93	-
9	rs12551834	131613191	G	A	0.09	IMPUTED	0.92	-
9	rs11788591	132502801	G	A	0.02	GENOTYPED	0.99	0.78
10	rs12722502	6093139	C	T	0.02	IMPUTED	0.96	-
10	rs943451	6621773	T	C	0.36	IMPUTED	0.88	-
10	rs10905284	8115362	C	A	0.42	GENOTYPED	1.00	0.96
10	rs11255753	8605553	G	T	-	-	-	-
10	rs2025758	8841669	T	C	0.46	IMPUTED	0.96	-
10	rs10508372	8972018	G	A	0.06	GENOTYPED	1.00	1.00
10	rs72782676	9032555	G	C	-	-	-	-
10	rs12413578	9049253	C	T	0.11	GENOTYPED	1.00	0.97
10	rs2893907	64382359	A	C	0.45	IMPUTED	0.93	-
10	rs75446656	65100016	C	A	0.05	IMPUTED	0.97	-
11	rs28415845	1145844	C	T	0.28	IMPUTED	0.96	-
11	rs10836538	36365253	G	T	0.36	IMPUTED	0.96	-
11	rs174535	61551356	T	C	0.35	GENOTYPED	1.00	0.99
11	rs479844	65551957	A	G	0.46	GENOTYPED	1.00	1.00
11	rs7130588	76270683	A	G	0.35	GENOTYPED	1.00	0.99
11	rs11236814	76343428	A	T	0.09	IMPUTED	0.94	-
11	rs1784775	111472460	T	C	0.31	IMPUTED	0.90	-
11	rs12365699	118743286	G	A	0.17	IMPUTED	0.96	-
11	rs56129466	128158189	A	G	0.24	IMPUTED	0.98	-
12	rs62623446	55368291	C	T	0.06	IMPUTED	0.71	-
12	rs10876864	56401085	G	A	0.39	GENOTYPED	1.00	0.99
12	rs3001426	57509055	T	C	0.47	IMPUTED	0.96	-
12	rs188074962	121365431	A	G	-	-	-	-
13	rs1319132	99999601	G	C	-	-	-	-

14	rs17103286	35885041	A	G	0.46	IMPUTED	0.97	-
14	rs3784099	68749927	G	A	0.29	IMPUTED	0.98	-
15	rs1655558	41787585	T	G	0.44	IMPUTED	0.95	-
15	rs11071559	61069988	C	T	0.13	GENOTYPED	1.00	0.95
15	rs117683492	67413624	G	A	0.02	IMPUTED	0.85	-
15	rs56375023	67448363	G	A	0.21	IMPUTED	0.99	-
15	rs34445740	67475764	NA	C	-	-	-	-
16	rs17806299	11199980	G	A	0.18	IMPUTED	0.99	-
16	rs12596613	11491007	C	G	0.33	IMPUTED	0.97	-
16	rs3024655	27369502	A	G	0.07	IMPUTED	0.84	-
16	rs2066844	50745926	C	T	0.05	GENOTYPED	1.00	0.90
17	rs146644295	37574592	G	C	0.02	IMPUTED	0.86	-
17	rs7216389	38069949	C	T	0.50	GENOTYPED	1.00	1.00
17	rs11658582	38763200	C	G	0.39	IMPUTED	0.93	-
17	rs112401631	38764524	T	A	0.01	IMPUTED	0.66	-
17	rs56308324	45819206	A	T	0.12	IMPUTED	0.99	-
17	rs17637472	47461433	G	A	0.41	IMPUTED	0.96	-
18	rs12965763	19067435	C	A	-	-	-	-
19	rs10413947	9128342	G	A	0.27	IMPUTED	0.83	-
19	rs10414065	33721455	C	T	0.06	IMPUTED	0.85	-
20	rs2766667	52172404	T	C	0.26	IMPUTED	0.92	-
20	rs1623866	62333022	G	A	0.23	IMPUTED	0.98	-
21	rs11088309	36464631	C	G	0.13	IMPUTED	0.98	-
22	rs2284033	37534034	G	A	0.43	GENOTYPED	1.00	0.99
22	rs5758364	41855912	C	A	-	-	-	-
23	rs850637	13023741	A	G	-	-	-	-
23	rs5953283	49139787	G	A	-	-	-	-

CHROM=chromosome, rsID=reference SNP cluster ID, POS=reference position, REF=reference allele, ALT=alternative non-reference allele, MAF=minor allele frequency in the specific cohort as the second most common allele count from the number of alleles in called genotypes in the specific cohort, TYPED=indicates weather the variant was genotyped or imputed, R2=imputation quality as the estimated value of the squared correlation between imputed genotypes and true/unobserved genotypes, ER2=empirical R2 for genotyped variants (not calculated for imputed variants)

Table S2. Information on 11 asthma-related single nucleotide polymorphisms that were included in our calculation of the polygenic risk score from the genome-wide association study by Ishigaki et al. [10].

					**Shika study**			
**CHROM**	**rsID**	**POS**	**REF**	**ALT**	**MAF**	**TYPED**	**R2**	**ER2**
2	rs4449174	242702538	C	G	0.17	IMPUTED	0.97	-
5	rs1837253	110401872	T	C	0.33	GENOTYPED	0.97	0.66
5	rs3857440	131794069	G	A	0.31	IMPUTED	0.99	-
6	rs1002045	29754015	G	A	0.19	IMPUTED	1.00	-
6	rs78218185	32432500	G	A	0.16	IMPUTED	0.96	-
9	rs10797119	92202495	T	C	0.31	IMPUTED	0.98	-
10	rs12253380	8940394	A	G	0.42	GENOTYPED	1.00	0.99
10	rs10795686	9045403	T	C	0.49	GENOTYPED	1.00	0.98
12	rs705704	56435412	G	A	0.18	IMPUTED	0.96	-
15	rs10519067	61068347	G	A	0.20	IMPUTED	0.97	-
16	rs3024577	27358203	A	G	0.29	IMPUTED	0.92	-

CHROM=chromosome, rsID=reference SNP cluster ID, POS=reference position, REF=reference allele, ALT=alternative non-reference allele, MAF=minor allele frequency in the specific cohort as the second most common allele count from the number of alleles in called genotypes in the specific cohort, TYPED=indicates weather the variant was genotyped or imputed, R2=imputation quality as the estimated value of the squared correlation between imputed genotypes and true/unobserved genotypes, ER2=empirical R2 for genotyped variants (not calculated for imputed variants)

Table S3. Description of the study samples, asthma and air pollution exposures in the main and sensitivity analyses.

	**Women with available asthma assessments (Table 2)**		**Women with available asthma assessments and information on genetics**		**Never-smoking women with available asthma assessments and information on genetics**	
	**German women: SALIA**	**Japanese women: Shika study**	**German women: SALIA**	**Japanese women: Shika study**	**German women: SALIA**	**Japanese women: Shika study**
**N**	771	847	532	410	432	343
**Diagnosed asthma (%)**	67 (8.69)	50 (5.90)	48 (9.02)	31 (7.56)	40 (9.26)	23 (6.71)
**Study characteristics**						
Mean age [years] ± sd	73.48 ± 3.05	67.00 ± 12.89	73.57 ± 2.96	60.26 ± 10.35	73.54 ± 2.95	61.36 ± 13.00
Mean height [cm] ± sd	163.19 ± 5.78	151.56 ± 6.80	163.04 ± 5.70	153.92 ± 5.83	162.91 ± 5.65	153.26 ± 7.40
Mean weight [kg] ± sd	72.52 ± 12.84	51.88 ± 8.44	72.65 ± 12.57	53.54 ± 7.75	72.30 ± 12.32	53.03 ± 9.00
<10 years education (%)	137 (17.77)	393 (46.40)	92 (17.29)	110 (26.83)	77 (17.82)	102 (29.74)
Ever-smoker (%)	150 (19.46)	97 (11.45)	100 (18.80)	67 (16.34)	0 (0)	0 (0)
**Air pollution exposures five years prior to the asthma assessments**						
Median $PM_{2.5}$ exposure [µg/m^3] (IQR)	17.36 (1.84)	12.70 (3.31)	17.32 (1.91)	11.65 (1.23)	17.27 (1.83)	11.65 (1.33)
Median NO_2 exposure [µg/m^3] (IQR)	25.93 (9.57)	8.54 (3.55)	25.37 (9.30)	7.30 (0.79)	24.86 (9.02)	7.30 (1.06)
sd= standard deviation, $PM_{2.5}$= particulate matter with an aerodynamic diameter ≤2.5µm, NO_2= nitrogen dioxide, IQR= interquartile range						

Table S4. Gene-environment interaction effects on asthma among German and Japanese women using different adjusted logistic regression models: the main results and results of sensitivity analyses.

Influencing factor [study sample-specific interquartile ranges]	**German women**			**Japanese women**		
	SALIA			Shika study		
	**Odds Ratio**	**95% confidence interval**	**p-value**	**Odds Ratio**	**95% confidence interval**	**p-value**
Analysis	**Main analysis: women with asthma and genetic information available<br>Adjusted for: age, height, weight, education, and ever-/never-smoking**					
N	**531**			**334**		
Polygenic risk score	0.54	0.346;0.848	0.007**	1.02	0.437;2.399	0.957
$PM_{2.5}$	1.19	0.789;1.810	0.401	18.42	5.869;57.824	<0.001**
**Polygenic risk score*$PM_{2.5}$**	1.22	0.660;2.273	0.520	1.17	0.427;3.185	0.764
Polygenic risk score	0.56	0.360;0.870	0.010*	1.44	0.752;2.749	0.273
NO_2	1.12	0.773;1.610	0.560	2.36	1.550;3.601	<0.001**
**Polygenic risk score*NO_2**	0.85	0.510;1.402	0.516	0.89	0.616;1.272	0.510
Analysis	**Repeated analysis: main analysis with binary polygenic risk [high-risk vs. low-risk group, not standardised in study sample-specific interquartile ranges] divided according to the median of continuous polygenic risk score<br>Adjusted for: age, height, weight, education, and ever-/never-smoking**					
N	**531**			**334**		
Polygenic high-risk vs. low-risk group	0.40	0.208;0.770	0.006**	1.28	0.374;4.377	0.694
$PM_{2.5}$	1.05	0.634;1.751	0.841	19.60	4.301;89.283	<0.001**
**Polygenic high-risk vs. low-risk group*$PM_{2.5}$**	1.31	0.573;3.012	0.519	0.93	0.177;4.885	0.932
Polygenic high-risk vs. low-risk group	0.41	0.214;0.786	0.007**	1.71	0.659;4.463	0.269
NO_2	1.17	0.773;1.755	0.465	2.65	1.495;4.691	<0.001**
**Polygenic high-risk vs. low-risk group*NO_2**	0.99	0.489;1.996	0.973	0.81	0.420;1.547	0.516
Analysis	**Repeated analysis: main analysis with applying the genetic risk score-interaction-training approach<br>Adjusted for: age, height, weight, education, and ever-/never-smoking**					
N test dataset	**355**			**223**		
Genetic risk score	1.04	0.695;1.562	0.842	0.91	0.309;2.686	0.865
$PM_{2.5}$	1.04	0.633;1.695	0.887	33.43	7.237;154.416	<0.001**
**Genetic risk score*$PM_{2.5}$**	1.30	0.793;2.124	0.299	3.03	0.776;11.806	0.111
Genetic risk score	1.22	0.870;1.702	0.251	2.01	0.966;4.182	0.062
NO_2	1.04	0.679;1.588	0.862	2.44	1.445;4.114	<0.001**
**Genetic risk score*NO_2**	1.07	0.700;1.641	0.749	1.13	0.626;2.027	0.691

Analysis	Stratified analysis: main analysis with excluding residential movers in the last 5 years before the asthma assessment<br>Adjusted for: age, height, weight, education, and ever-/never-smoking					
N	**520**					
Polygenic risk score	0.63	0.408;0.987	0.043*	[The Shika study only included individuals that lived in the Shika town at the time of examination, so that residential movers between the examinations were automatically excluded]		
$PM_{2.5}$	1.14	0.754;1.714	0.540			
**Polygenic risk score*$PM_{2.5}$**	1.38	0.784;2.417	0.266			
Polygenic risk score	0.65	0.422;1.009	0.054			
NO_2	1.14	0.806;1.599	0.467			
**Polygenic risk score*NO_2**	1.01	0.632;1.625	0.957			
Analysis	**Stratified analysis: main analysis with excluding ever-smoker women<br>Adjusted for: age, height, weight, and education**					
N	**431**			**274**		
Polygenic risk score	1.20	0.774;1.845	0.420	0.97	0.332;2.858	0.961
$PM_{2.5}$	1.21	0.781;1.865	0.396	23.08	5.543;96.137	<0.001**
**Polygenic risk score*$PM_{2.5}$**	0.59	0.315;1.095	0.094	1.07	0.296;3.895	0.914
Polygenic risk score	1.19	0.767;1.836	0.441	1.36	0.61;3.009	0.455
NO_2	1.10	0.768;1.586	0.594	2.89	1.602;5.208	<0.001**
**Polygenic risk score*NO_2**	0.79	0.472;1.306	0.352	0.83	0.480;1.421	0.489
$PM_{2.5}$= particulate matter with an aerodynamic diameter of ≤2.5µm, NO_2= nitrogen dioxide, *= p-value<0.05, **= p-value<0.01						

Figures

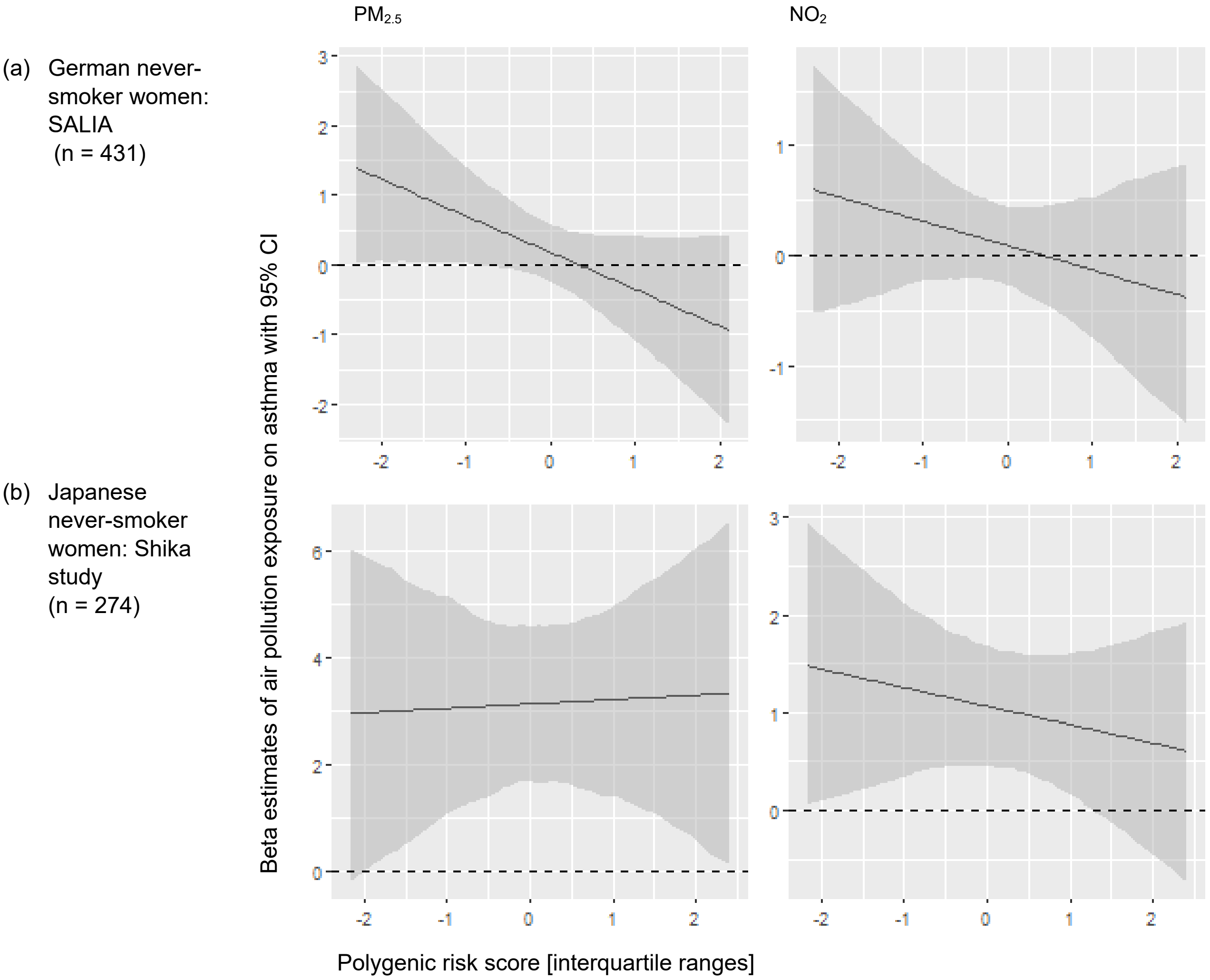

Figure S1. Effects of air pollution exposure on asthma for each sample-specific interquartile range increase of the polygenic risk score in elderly (a) German never-smoker women and (b) Japanese never-smoker women.

CI= confidence interval, $PM_{2.5}$= particulate matter with an aerodynamic diameter ≤2.5µm, NO_2= nitrogen dioxide
Adjusted for: age, height, weight, and education

References

1. Beelen, R.; Hoek, G.; Vienneau, D.; Eeftens, M.; Dimakopoulou, K.; Pedeli, X.; Tsai, M.-Y.; Künzli, N.; Schikowski, T.; Marcon, A.; et al. Development of NO2 and NOx land use regression models for estimating air pollution exposure in 36 study areas in Europe – The ESCAPE project. Atmospheric Environment **2013**, 72, 10–23, doi:10.1016/j.atmosenv.2013.02.037.
2. Eeftens, M.; Beelen, R.; Hoogh, K. de; Bellander, T.; Cesaroni, G.; Cirach, M.; Declercq, C.; Dėdelė, A.; Dons, E.; Nazelle, A. de; et al. Development of Land Use Regression models for PM(2.5), PM(2.5) absorbance, PM(10) and PM(coarse) in 20 European study areas; results of the ESCAPE project. *Environ. Sci. Technol.* **2012**, *46*, 11195–11205, doi:10.1021/es301948k.
3. Kawai, Y.; Mimori, T.; Kojima, K.; Nariai, N.; Danjoh, I.; Saito, R.; Yasuda, J.; Yamamoto, M.; Nagasaki, M. Japonica array: improved genotype imputation by designing a population-specific SNP array with 1070 Japanese individuals. *J. Hum. Genet.* **2015**, *60*, 581–587, doi:10.1038/jhg.2015.68.
4. Reed, E.; Nunez, S.; Kulp, D.; Qian, J.; Reilly, M.P.; Foulkes, A.S. A guide to genome-wide association analysis and post-analytic interrogation. *Stat. Med.* **2015**, *34*, 3769–3792, doi:10.1002/sim.6605.
5. Das, S.; Forer, L.; Schönherr, S.; Sidore, C.; Locke, A.E.; Kwong, A.; Vrieze, S.I.; Chew, E.Y.; Levy, S.; McGue, M.; et al. Next-generation genotype imputation service and methods. *Nat. Genet.* **2016**, *48*, 1284–1287, doi:10.1038/ng.3656.
6. Song, S.; Jiang, W.; Hou, L.; Zhao, H. Leveraging effect size distributions to improve polygenic risk scores derived from summary statistics of genome-wide association studies. *PLoS Comput. Biol.* **2020**, *16*, e1007565, doi:10.1371/journal.pcbi.1007565.
7. Hüls, A.; Krämer, U.; Carlsten, C.; Schikowski, T.; Ickstadt, K.; Schwender, H. Comparison of weighting approaches for genetic risk scores in gene-environment interaction studies. *BMC Genet.* **2017**, *18*, 115, doi:10.1186/s12863-017-0586-3.
8. Papi, A.; Brightling, C.; Pedersen, S.E.; Reddel, H.K. Asthma. *The Lancet* **2018**, *391*, 783–800, doi:10.1016/S0140-6736(17)33311-1.
9. El-Husseini, Z.W.; Gosens, R.; Dekker, F.; Koppelman, G.H. The genetics of asthma and the promise of genomics-guided drug target discovery. *The Lancet Respiratory Medicine* **2020**, *8*, 1045–1056, doi:10.1016/S2213-2600(20)30363-5.
10. Ishigaki, K.; Akiyama, M.; Kanai, M.; Takahashi, A.; Kawakami, E.; Sugishita, H.; Sakaue, S.; Matoba, N.; Low, S.-K.; Okada, Y.; et al. Large-scale genome-wide association study in a Japanese population identifies novel susceptibility loci across different diseases. *Nat Genet* **2020**, *52*, 669–679, doi:10.1038/s41588-020-0640-3.
